# *In Vitro* Anti-diabetic Activities and Chemical Analysis of Polypeptide-k and Oil Isolated from Seeds of *Momordica charantia* (Bitter Gourd)

**DOI:** 10.3390/molecules17089631

**Published:** 2012-08-10

**Authors:** Zuraini Ahmad, Khairul Faizi Zamhuri, Azhar Yaacob, Chiong Hoe Siong, Malarvili Selvarajah, Amin Ismail, Muhammad Nazrul Hakim

**Affiliations:** 1 Department of Biomedical Sciences, Universiti Putra Malaysia, 43400 UPM Serdang Selangor, Malaysia; Email: zuraini@medic.upm.edu.my (Z.A.); k_ag88@yahoo.com (K.F.A.); chionghs@yahoo.com (C.H.S.); vilisha_raj@hotmail.com (M.S.); 2 Sports Academy, Universiti Putra Malaysia, 43400 UPM Serdang Selangor, Malaysia; Email: art2azhar@yahoo.com; 3 Department of Nutrition and Dietetics, Universiti Putra Malaysia, 43400 UPM Serdang Selangor, Malaysia; Email: amin@medic.upm.edu.my

**Keywords:** *Momordica charantia*, amino acids, fatty acids, α-glucosidase, α-amylase

## Abstract

The amino acid and fatty acid composition of polypeptide k and oil isolated from the seeds of *Momordica charantia* was analysed. The analysis revealed polypeptide k contained 9 out of 11 essential amino acids, among a total of 18 types of amino acids. Glutamic acid, aspartic acid, arginine and glycine were the most abundant (17.08%, 9.71%, 9.50% and 8.90% of total amino acids, respectively). Fatty acid analysis showed unusually high amounts of C18-0 (stearic acid, 62.31% of total fatty acid). C18-1 (oleic acid) and C18-2 (linoleic acid) were the other major fatty acid detected (12.53% and 10.40%, respectively). The oil was devoid of the short fatty acids (C4-0 to C8-0). Polypeptide k and oil were also subjected to *in vitro* α-glucosidase and α-amylase inhibition assays. Both polypeptide k and seed oil showed potent inhibition of α-glucosidase enzyme (79.18% and 53.55% inhibition, respectively). α-Amylase was inhibited by 35.58% and 38.02%, respectively. Collectively, the *in vitro* assay strongly suggests that both polypeptide k and seed oil from *Momordica charantia *are potent potential hypoglycemic agents.

## 1. Introduction

Approximately 60–80% of the World’s population relies on plant-based medicines as their health care system. The traditional Indian system of medicine (Ayurveda) has a long history, however many of the plants used lack any adequate scientific documentation to support this use. Nevertheless, it is now clear that the medicinal value of these plants lies in the bioactive phytochemical constituents that produce definite biomedical effects on the human body [1]. *Momordica charantia* Linn (MC) or bitter gourd is one such plant that has been extensively used and reported for its hypoglycemic effects [2,3]. It has been commonly used as a traditional remedy in Asia, Africa, England, India and Sri Lanka [4]. In Ayurveda, the fruit is considered as to have many benifits such as tonic, stimulant, emetic, antibilous, laxative and alterative. However, some have claimed that MC will induce heartburn and worsen gastric ulcers [5]. 

MC contains several very promising bioactive compounds that activate adenosine monophosphate-activated protein kinase (AMPK), which is well known for regulating energy metabolism and enabling glucose uptake which is impaired in diabetics [5]. MC also contains a lectin that has insulin-like activity due to its affinity to the insulin receptors. This compound lowers blood glucose concentrations by acting on peripheral tissues and suppressing appetite [6]. Fruit and seeds of MC are traditionally used as a medicinal herb and/or vegetable for treatment of diabetes in Southeast Asian countries [7]. Its *in vivo* hypoglycemic activity has been reported for pulps, seeds and leaves [8]. Khanna *et al*. [9] isolated an active protein (polypeptide-p) from seeds by acid ethanol extraction, which decreased blood glucose in Streptozotocin-induced diabetic rats and increased glycolytic enzyme activity. Polypeptide-p has also shown to have hypoglycemic effects in juvenile and maturity-onset diabetic patients [9,10]. Polypeptide-k (PPK), which was later isolated from seeds of MC, also possesses blood glucose level-reducing activity which is more potent than that of polypeptide-p and helps in the prevention of diabetes [11]. Research and clinical trials have proven that this product can sucessfully regulate blood glucose [12]. The oil from the seeds of MC also has biomedical properties as reported by Noguchi *et al*. [13] and Braca *et al*. [14].

α-Glucosidase (EC 3.2.1.20, α-D-glucoside glucohydrolase) is an exo-carbohydrase that catalyzes the liberation of α-glucose from carbohydrates. This enzyme is widely distributed in microorganisms, plants, and animal tissues [15]. α-Amylase (EC 3.2.1.1) is an enzyme that hydrolyses the α-bonds of large α-linked polysaccharides. Found in many tissues, amylase is most prominent in pancreatic juice and saliva [16]. Thus, the objectives of this current investigation were to determine the chemical properties of PPK and oil isolated from MC seeds and determine their potential anti-diabetic activity *in vitro *through investigating their effects on inhibition of α-glucosidase and α-amylase.

## 2. Results and Discussion

The fatty acid analysis of MC seed oil is shown in Table 1. The most abundant fatty acid in MC seed oil is C18-0, followed by C18-1 and C18-2. The profile is different when compared to the common palm oil which contains predominantly C16-0 and C18-1. Both oils are devoid of short chain fatty acids (C4-0 to C8-0). The amino acid composition of PPKis shown in Table 2. The major amino acids are glutamic acid, aspartic acid, arginine and glycine (ranging from 8.9 to 17.08% of the total). Table 3 shows the anti-oxidant activity of polypeptide-k and MC seed oil. Standard α-tocopherol has the highest anti-oxidant activity of 96.58% when compared to PPK and MC seed oil (84.9 and 80.85% respectively). Palm oil displayed the lowest anti oxidant activity.

**Table 1:** Fatty acid composition of oil from seeds of *Momordica charantia* (MCOP) and palm oil (PO, control).

	MCO	PO
C4-0 (Butyric acid)	ND	ND
C6-0 (Caprioc acid)	ND	ND
C8-0 (Caprylic acid)	ND	ND
C10-0 (Capric acid)	0.53 ± 0.03	ND
C12-0 (Lauric acid)	0.45 ± 0.04	0.3 ± 0.12
C14-0 (Myristic acid)	ND	1.1 ± 0.02
C14-1 (Myristoliec acid)	ND	ND
C16-0 (Palmitic acid)	4.72 ± 0.28	43.7 ± 1.95
C16-1 (Palmitoleic acid)	ND	0.5 ± 0.05
C18-0 (Stearic acid)	62.31 ± 3.74	4.9 ± 0.18
C18-1 (Oleic acid)	12.53 ± 0.70	37.8 ± 0.94
C18-2 (Linoleic acid)	10.40 ± 0.63	10.1 ± 1.0
C20-0 (Arachidic acid)	5.30 ± 0.39	0.43 ± 0.07
Others	2.76 ± 0.18	0.45 ± 0.16

Values are % of total fatty acid expressed as mean ± SD of three separate determinations. ND: not detected.

The chemical analysis of two constituents from the seeds of MC was investigated. The MC seed oil contains high amount of stearic acid (C18-0). This is very interesting as MC seed oil has been reported to posses wound healing and anti-diabetic properties [17]. Long chain saturated fatty acids are not recommended for human ingestion, but Bonanome and Grundy [18] reported reduction of plasma lipoprotein levels in 11 subjects during three dietary periods when given stearic acid. PPK isolated also from the seeds of MC has been reported to have potent anti-diabetic activity. It has been given to diabetic patients with good results [11]. In our previous study [12], we reported polypeptide k supplemented rolls accelerate the reduction of blood glucose in normal healthy individuals. As PPK contains a balanced amount or all amino acids, it can be a useful ingredient for many food, *i.e.*, bread, noodles, flour, biscuits, *etc.* Indeed both MC seed oil and PPK had potent anti-oxidant properties which may be beneficial in reducing β-cell damage [11,12], thus improving the severity of diabetes. Interestingly, these results may be part of the key mechanism for the reduction of blood glucose observed in the clinical trials done previously, when 605 diabetic patients were administered PPK sublingually for 1 to 3 years for best effects (avoiding the first-pass metabolism effects) and blood glucose was reduced approximately 25 to 43.6% [11]. This study also revealed that patients taking PPK orally also had a reduction in elevated blood glucose [11]. Indeed, this product has been in Malaysian market for the past 10 years with very effective results.

**Table 2:** Amino acid composition of polypeptide-k isolated from *Momordica charantia*.

Amino acid	Polypeptide-k
Aspartic acid	9.71 ± 0.43
Glutamic acid	17.08 ± 0.95
Serine	5.3 ± 0.15
Glycine	8.9 ± 0.57
Histidine	3.4 ± 0.23
Arginine	9.5 ± 1.57
Threonine	3.0 ± 0.01
Alanine	7.6 ± 1.21
Proline	4.1 ± 0.20
Tyrosine	2.7 ± 0.14
Valine	5.8 ± 0.35
Methionine	1.6 ± 0.25
Cystine	2.8 ± 0.23
Isoleucine	4.8 ± 0.12
Leucine	3.7 ± 0.28
Phenylalanine	4.2 ± 0.19
Lysine	4.4 ± 0.36
Tryptophan	1.0 ± 0.02

Values are % of total amino acid expressed as mean ± SD of three separate determinations.

PPK contains nine out of 11 essential amino acids, making it a good source of amino acids [19]. It also contains high amount of glutamic acid, glycine and arginine which are vital for many bodily functions. Glutamic acid and glycine are important for memory, learning and neurotransmitter [20]. Arginine plays an important role in blood circulation, healing of wounds, treatment of erectile dysfunction and immune functions [21]. Table 3 reveals that PPK and seed oil have potent anti-oxidant activity that may help in the reduction of blood glucose. 

**Table 3:** Total anti-oxidant activity of polypeptide-k and oil from *Momordica charantia.*

Sample	Percentage anti oxidant activity (%)
α-Tocopherol (Standard)	96.58 ± 1.76
MCO	80.54 ± 2.41
PPK	84.90 ± 1.71
PO	45.21 ± 6.90

Values are % of antioxidant expressed as mean ± SD of three separate determinations. MCO: *Momordica charantia* seed oil. PPK: Polypeptide-k. PO: palm oil.

The MC seed oil also contains many other phytocompounds that may have other biomedical properties. The oil contain high amount of *trans*-nerolidol, *cis*-dihydrocarveol and apiole [14]. Recently, Pripdeevech and Machan [22] reported potent anti-oxidant properties in different teas in Thailand and *trans*-nerolidol is one of the many volatile components identified. *cis*-Dihydrocarveol and apiole are related to anti-microbial activity [14]. Interestingly, seed oil also has the potential as it contains trans-Nerolidol and Apiole (2 major constituents) which are anti-microbial and help in wound healing [17].

**Figure 1:**
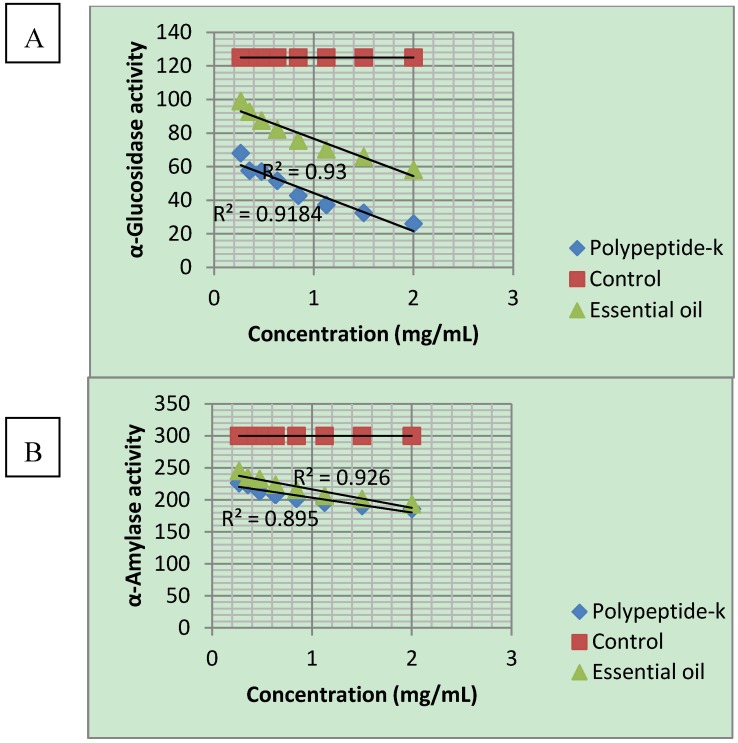
Dose-dependent inhibition of α-glucosidase and α-amylase activities by polypeptide-k and Momordica charantia seed oil. A: α-glucosidase; B: α-amylase.

The α-glucosidase inhibition is expressed in Table 4. PPK has the highest inhibition of 79.18% at 2 mg/mL as compared to MC seed oil of 53.55% at the same concentration. This inhibition was in a dose-dependent manner (Figure 1). The inhibition of α-amylase is illustrated in Table 5. Both PPK and MC seed oil inhibited the α-amylase activity from 18.20 to 35.58% and 24.54 to 38.02%, respectively. MC seed oil has slightly higher inhibition when compared to PPK. Inhibition of α-amylase also occurred in a dose-dependent manner (Figure 1).

**Table 4:** Effect of polypeptide-k and oil from *Momordica charantia* on α-glucosidase activity.

Concentration (mg/mL)	PPK α-Glucosidase activity (% Inhibition)	MCO α-Glucosidase activity (% Inhibition)
0.267	70.6628 ± 5.0856 (45.55)	97.0091 ± 1.0726 (20.71)
0.356	51.4710 ± 9.9259 (53.89)	90.7401 ± 1.1301 (25.84)
0.475	55.4295 ± 2.9121 (54.33)	85.6295 ± 3.0594 (30.09)
0.633	48.5088 ± 3.6516 (58.73)	80.4882 ± 3.4838 (34.30)
0.844	42.7553 ± 4.6282 (65.79)	74.0152 ± 2.1990 (39.55)
1.125	37.2303 ± 4.8153 (70.21)	68.8970 ± 2.5743 (43.75)
1.5	32.4798 ± 2.0861 (74.01)	64.5213 ± 2.0793 (47.31)
2	26.0133 ± 2.5803 (79.18)	56.8900 ± 2.9721 (53.55)

All values are mean ± SD, n = 8.

**Table 5:** Effect of polypeptide-k and oil from *Momordica charantia* on α-amylase activity.

Concentration (mg/mL)	PPK α-Amylase activity (% Inhibition)	MCO α-Amylase activity (% Inhibition)
**0.267**	226.3502 ± 9.3639 (18.20)	245.3911 ± 9.8317 (24.54)
**0.356**	223.3028 ± 9.3302 (21.54)	235.3548 ± 12.4998 (25.56)
**0.475**	213.3901 ± 8.4586 (22.42)	232.7123 ± 12.9344 (28.86)
**0.633**	208.0655 ± 9.1613 (25.43)	223.6822 ± 12.3970 (30.64)
**0.844**	201.9705 ± 9.0938 (28.09)	215.7165 ± 12.3385 (32.67)
**1.125**	195.8756 ± 9.0263 (31.10)	206.6906 ± 13.2289 (34.70)
**1.5**	190.5341 ± 8.2053 (32.88)	201.3527 ± 11.8301 (36.48)
**2**	185.9291 ± 5.1072 (35.58)	193.2560 ± 7.6110 (38.02)

All values are mean ± SD, n = 8.

One therapeutic strategy for the treatment of diabetes is to decrease hyperglycemia post-ingestion. This can be done by inhibiting carbohydrate-hydrolyzing enzymes such as α-amylase and α-glucosidase [23]. In this current study, we demonstrated a large inhibition of 79% for α-glucosidase and 38% for α-amylase, suggesting that both polypeptide k and MC seed oil are potential potent anti-diabetic agents. By inhibiting these enzymes, they will delay carbohydrate digestion and prolong the overall time for carbohydrate digestion resulting in a reduction in the rate of glucose absorption and consequently blunting the post-parandial blood glucose rise [24], *i.e.*, making the food lower in the glycemic index.

Previously, Ali *et al*. [23] reported several Malaysian plants (*Phyllantus spp*.) inhibited α-amylase activity by 24%, which they reported to be potent inhibition. In this study, we reported more potent inhibition of 35.58% and 38.02% for PPK and MC seed oil respectively. Interestingly, α-glucosidase revealed even bigger inhibition. Babu *et al.* [25] reported approximately 35% inhibition of Himalayan herbs constituents which are used in treating diabetes when compared to 79% in this current study. The inhibition of these enzymes may be the other key factors in the mechanism for the effects of PPK observed clinically [11,12]. The major setback of commercial anti-diabetic drugs is hypoglycemia if taken more than the recommended dose. Interestingly, *in vivo *in humans, no hypoglycemic effects were observed even when patients took more than the recommended dose [11]. This is the major advantage of PPK over all other commercial anti-diabetic drugs. 

## 3. Experimental

### 3.1. General

Polypeptide-k and oil from seeds of MC were generous gifts from Magna Bio-Laboratories Sdn. Bhd. Malaysia (Batch 37/11, 121/11 and 375/11). Voucher specimens were deposited in the herbarium of the Institute of Bioscience, UPM, Malaysia. Polypeptide-k was isolated using the method of Kanna [11] and oil was isolated using methods of Noguchi *et al*. [13] and Kanna [17]. In this case, we used ripe bitter melon approximately 14–20 days after fruiting (3–3.5 months after planting). Other chemicals are of analytical grades and were obtained from Sigma Chemicals (St. Louis, MO, USA).

### 3.2. Antioxidant Activity

The antioxidant activity of extract was evaluated by the β-carotene-linoleate assay as described by Amin and Tan [26]. Briefly, β-carotene solution (1.0 mL, 0.2 mg/mL in chloroform) was pipetted into a round bottom flask (250 mL) containing linoleic acid (0.02 mL) and 100% Tween 20 (0.2 mL). The mixture was evaporated at 40 °C for 10 min and diluted with distilled water (100 mL) slowly and agitated vigorously to form an emulsion. Five mL aliquots of the emulsion were transferred into different test tubes containing 0.2 mL of samples in solvents at a final concentration of 1 mg/mL. The mixture was then gently shaken and placed in water bath at 45 °C for 2 h. The absorbance of the samples was measured at 470 nm using a spectrophotometer (UV-1610, Shimadzu, Kyoto, Japan) at initial time (t = 0) against a blank, consisting of an emulsion without β-carotene. Standard (α-tocopherol) of the same concentration of samples were used for comparison. A 0.2-mL amount of methanol in 5 mL of the above emulsion was used as the control. Measurements were carried out at 15 min intervals for 120 min. The antioxidant activity (AA) was measured in terms of successful bleaching of β-carotene.

### 3.3. Fatty Acid Composition of the Seed Oil

Total lipid extraction of the samples was carried out in triplicate as described previously [27,28], using a chloroform/methanol (2:1, v/v) solvent system. Transmethylation was carried out using 14% methanolic boron trifluoride. The derived fatty acid methyl esters (FAMEs) were separated on a Quadrex 007 series bonded phase fused silica capillary column (Quadrex Corporation, New Haven, CT, USA) (30 m × 0.25 mm ID, 0.20 mm film thickness, 007 Carbowax/BTR) in a 5890 Hewlett-Packard Gas-Liquid Chromatograph (Hewlett-Packard Co., Avondale, PA, USA). Individual fatty acids were identified and quantified by comparison with retention times and peak areas of FAMEs standards from Supleco Inc. (Bellefonte, PA, USA). Commercial palm oil from Malaysia was used as reference.

### 3.4. Amino Acid Determination of Polypeptide-k

The methods of Vidotti *et al*., [29] were employed, as previously modified [27,28]. Triplicate samples were hydrolysed with 6 N hydrochloric acid for 24 h at 110 °C. The hydrolysed samples were then analysed using an automatic amino acid analyzer L-8500 (Hitachi, Tokyo, Japan) with a ninhydrin reagent and lithium buffer system by injecting 20 μL [30]. The reproducibility of the results was within approximately 3%. The net height of each peak produced by the chart recorder of the analyzer (each representing an amino acid) was measured and calculated.

### 3.5. α-Glucosidase Inhibition

α-Glucosidase activity was measured using a protocol based on Ye *et al*. [16] with the following modifications: the inhibitory effect of the compounds on α-glucosidase were assessed by using 4-nitrophenyl-α-D-glucopyranoside (PNPG) as substrate (0.2 mL 20 mmol·L^−1^). Reaction system: 2.0 mL 0.1 mol·L^−1^ potassium phosphate buffer (pH 6.8), 0.1 mL sample solution, 50 μL reduced glutathione (1 mg·mL^−1^), 0.1 mL α-glucosidase solution (0.57 U·mL^−1^), which were well mixed, after being pre-incubated for 15 min at 37 °C, 0.2 mL of PNPG (20 mmol·L^−1^) was added, 37 °C for 15 min, after stopping the reaction by addition of 10 mL of 0.1 mol·L^−1^ Na_2_CO_3_. The amount of 4-nitrophenol was measured spectrophotometrically at 400 nm. 

### 3.6. α-Amylase Inhibition

The α-amylase inhibition assay was performed using the chromogenic method adopted from Sigma–Aldrich, as adapted from Ali *et al*., [23]. Bovine pancreatic α-amylase (EC 3.2.1.1, Sigma) was dissolved in ice-cold distilled water to give a concentration of 4 unit/mL solution. Potato starch (0.5%, w/v) in 20 mM phosphate buffer (pH 6.9) containing 6.7 mM sodium chloride, was used as a substrate solution. α-Amylase activity was determined by measuring the absorbance of the mixture at 540 nm. Control incubations, representing 100% enzyme activity were conducted in an identical fashion replacing compound/oil with DMSO.

## 4. Conclusions

Collectively, the results from this present study strongly suggest that both polypeptide k and MC seed oil have potent anti-diabetic activity *in vitro* and also high anti-oxidant activity. Furthermore, oil from MC seed oil has the potential for wound healing [17] and polypeptide-k contains balanced amino acid which is suitable to be used as a source for amino acids in processed food.
